# Extra Virgin Olive Oil Extracts of Indigenous Southern Tuscany Cultivar Act as Anti-Inflammatory and Vasorelaxant Nutraceuticals

**DOI:** 10.3390/antiox11030437

**Published:** 2022-02-22

**Authors:** Luca Pozzetti, Francesca Ferrara, Ludovica Marotta, Sandra Gemma, Stefania Butini, Mascia Benedusi, Fabio Fusi, Amer Ahmed, Serena Pomponi, Stefano Ferrari, Matteo Perini, Anna Ramunno, Giacomo Pepe, Pietro Campiglia, Giuseppe Valacchi, Gabriele Carullo, Giuseppe Campiani

**Affiliations:** 1 Department of Biotechnology, Chemistry and Pharmacy, DoE 2018–2022, University of Siena, 53100 Siena, Italy; luca.pozzetti@student.unisi.it (L.P.); ludovica.marotta@student.unisi.it (L.M.); gemma@unisi.it (S.G.); butini3@unisi.it (S.B.); fabio.fusi@unisi.it (F.F.); 2 Department of Neuroscience and Rehabilitation, University of Ferrara, 44121 Ferrara, Italy; frrfnc3@unife.it (F.F.); bndmsc@unife.it (M.B.); 3 Department of Life Sciences, University of Siena, 53100 Siena, Italy; aa.biotechiub@gmail.com; 4 Società Agricola Olivicoltori delle Colline del Cetona Società Cooperativa, 53100 Siena, Italy; info@frantoiodelcetona.it; 5 ISVEA s.r.l., 53036 Poggibonsi (SI), Italy; s.ferrari@isvea.it; 6 Fondazione Emund Mach, 38098 San Michele all’Adige (TN), Italy; matteo.perini@fmach.it; 7 Department of Pharmacy, University of Salerno, 84084 Fisciano (SA), Italy; aramunno@unisa.it (A.R.); gipepe@unisa.it (G.P.); pcampiglia@unisa.it (P.C.); 8 Department of Animal Science, Plants for Human Health Institute, NC State University, Kannapolis, NC 28081, USA; gvalacc@ncsu.edu; 9 Department of Environmental Sciences and Prevention, University of Ferrara, 44121 Ferrara, Italy; 10 Department of Food and Nutrition, Kyung Hee University, Seoul 02447, Korea

**Keywords:** olive oil, traceability quality, food origin, luteolin, oleuropein, CaCo-2 cells, Nrf2, NF-κB

## Abstract

Extra virgin olive oil (EVOO) is the typical source of fats in the Mediterranean diet. While fatty acids are essential for the EVOO nutraceutical properties, multiple biological activities are also due to the presence of polyphenols. In this work, autochthonous Tuscany EVOOs were chemically characterized and selected EVOO samples were extracted to obtain hydroalcoholic phytocomplexes, which were assayed to establish their anti-inflammatory and vasorelaxant properties. The polar extracts were characterized via ^1^H-NMR and UHPLC-HRMS to investigate the chemical composition and assayed in CaCo-2 cells exposed to glucose oxidase or rat aorta rings contracted by phenylephrine. Apigenin and luteolin were found as representative flavones; other components were pinoresinol, ligstroside, and oleuropein. The extracts showed anti-inflammatory and antioxidant properties via modulation of NF-κB and Nrf2 pathways, respectively, and good vasorelaxant activity, both in the presence and absence of an intact endothelium. In conclusion, this study evaluated the nutraceutical properties of autochthonous Tuscany EVOO *cv.*, which showed promising anti-inflammatory and vasorelaxant effects.

## 1. Introduction

The Mediterranean diet (MD) is nowadays considered a philosophy of life, in addition to being a beneficial lifestyle [1]. This diet limits the incidence rates of cardiovascular and neurodegenerative diseases, cancer, and metabolic disorders associated with reactive oxygen species (ROS) [2]. Since MD is based on the typical autochthonous fauna and flora of the Mediterranean countries [3], the typical source of fats is represented by extra virgin olive oil (EVOO) [4].

Among the bioactive compounds present in EVOO, the non-saponifiable fraction is constituted by lipophilic compounds [5], such as tocopherols, carotenoids, lutein, and carotene. EVOO also contains more hydrophilic molecules, namely phenolic compounds, such as secoiridoids [6], which contribute to its bitter taste, thereby making it highly appreciated [7]. The polar composition of the EVOO may depend on the cultivar, ripening, and harvesting methods, but also on technological parameters, such as milling, malaxation, separation phases, and storage or distribution factors [8].

The consumption of 30–50 g/day of EVOO is able to prevent many pathologies [9]. EVOO has been frequently reported to have anti-inflammatory and immunomodulatory activities and to reduce the risk of coronary heart disease by modulating high-density lipoprotein (HDL) and cholesterol metabolism [10]. Seminal studies have shown improvements in renal function, lipid profile, oxidative stress [11], and inflammatory parameters in patients treated with EVOO (40 mL/day) for 9 weeks [12].

Notably, EVOO is also able to improve endothelial functions [13], and these positive effects are due, at least in part, to the presence of hydrophilic components such as biophenols, which are well-recognized for their remarkable antioxidant activity [14,15,16].

After ingestion, the edible phenols present in EVOO should be absorbed by enterocytes to reach the target organs and exert their biological activity. Nevertheless, when biophenols are consumed within the diet, their absorption is limited by interactions with food matrices and by the actions of digestive enzymes [17].

Interestingly, recent literature data demonstrated that a combination of phytochemicals, such as physical mixture or complex matrices, was responsible for better biological effects [18]. Specifically, EVOO hydroalcoholic extracts mediated vasorelaxation in rat mesenteric arteries by activating potassium channels through an increase in intracellular [Ca^2+^] [19]. EVOO extracts decreased inflammation in dendritic cells by reducing tumor necrosis factor-α (TNF-α) and interleukin 6 (IL-6) secretion with downregulation of intracellular nitric oxide synthase (iNOS) expression [20].

In this context, we investigated EVOO extracts from olive oils produced in an Italian area highly suited to Protected Denomination of Origin (PDO) EVOO production, collected in a consortium namely Consorzio Olivicoltori delle Colline del Cetona scarl (Siena, Tuscany, Italy), an area which spans from the plains to the hills at the slope of Cetona mountain, characterized by a mild climate that is perfect for the farming of different cultivars of olive trees, including indigenous cultivars such as *Leccino* and *Minuta di Chiusi*. The latter is a rare and ancient olive cultivar, which flourishes only in this part of South Tuscany. This *cv.*, which is different from the more famous *Minuta* cultivars from Sicily and Calabria, shares with them a high resistance to low temperatures and to the olive fruit fly (*Bactrocera oleae*) [21].

This *Minuta* olive oil is very delicate and rich in subtle fruity notes with floral aromas; to the best of our knowledge, this is the first attempt at promoting the biological properties of this specific *cv.* grown in Tuscany.

We report here the chemical characterization of 18 EVOO samples in terms of acidity, peroxide values, tocopherols, biophenols, and polyphenols. Based on these results, we further selected three representative samples (**O_05**, **O_10**, and **O_15**) based on the rare, ancient, and indigenous cultivar *Minuta di Chiusi* (**O_15**) and the indigenous monocultivar *Leccino* (**O_10**), while **O_05** derives from different cultivars (*Leccino*, *Frantoio*, *Morello*, *Moraiolo,* and *Greggiolo*). Liquid–liquid extraction was performed to obtain hydroalcoholic extracts (**OE**), which were then submitted to ^1^H NMR and UHPLC-HRMS experiments for qualitative characterization in terms of bioactive phenols. Then, we evaluated the antioxidant and anti-inflammatory properties of EVOO polar extracts in human intestinal colorectal cells (CaCo-2 cells), where an oxidative stress stimulus was induced by the exposure to the enzyme glucose oxidase (GO). The EVOO extracts were also analyzed to assess their vasorelaxation potential in rat aorta rings. Overall, the extracts promoted a good antioxidant and anti-inflammatory response in the CaCo-2 cell line by modulating the expression of specific inflammatory mediators such as Nrf2, IL-6, and iNOS. When tested in both endothelium-intact and -denuded rat aorta rings, **OE_15** showed significant vasorelaxation, while in denuded aorta rings **OE_05** and **OE_10** proved to be worthy vasorelaxant agents. Thus, **OE_05** and **OE_10** showed nutraceutical potential, suggesting their beneficial effects in alleviating endothelial dysfunction. On the other hand, **OE_15** could be proposed as a nutraceutical supplement to prevent hypertension.

## 2. Materials and Methods

### 2.1. General Chemical Considerations

All chemicals and solvents were purchased from Merck (Milan, Italy) as HPLC grade; the compounds used as internal or external standards were purchased from Merck (Milan, Italy) as analytical standard grade.

### 2.2. EVOO Samples

**Figure 1:**
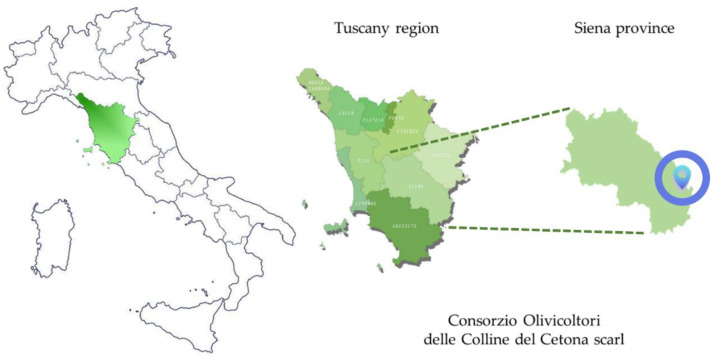
Geographical distribution of the samples of EVOO.

EVOO samples were collected from different farms located in a small area of the southern province of Siena, Tuscany. The olive trees from different *cv.*, including *Frantoio, Leccino, Minuta di Chiusi, Moraiolo, Greggiolo,* and others, were grown at different altitudes, in a range between 300 and 600 m above sea level. The fruits were collected between 20 October 2020 and 26 November 2020 and cold-milled no more than 12 h after the harvest at the olive oil mill “Olivicoltori delle Colline del Cetona S.c.a.r.l.”, S.S. 321 km 11, 53040 Cetona, Siena, Tuscany, Italy (Figure 1). The samples were collected on the same day of the milling and stored in dark glass bottles at 4 °C for no more than 3 days before the start of the analyses.

### 2.3. Determination of Free Fatty Acids

The free fatty acid content was determined following the Commission Regulation (EEC) No. 2568/91 of 11 July 1991 [22]. Briefly, an exactly weighted amount of about 10 g of EVOO sample was solubilized in 100 mL of a freshly prepared solution of ethanol and diethyl ether (1:1 ratio), neutralized with a solution of KOH 0.1 N in ethanol, with the addition of 0.3 mL of phenolphthalein solution (10 g/L in absolute ethanol) as an indicator. The sample solution was then titrated while stirring with the KOH solution until the indicator changed. The acidity value, expressed as a percentage of oleic acid by weight, was the result of an arithmetic mean between two determinations from the same sample of olive oil.

### 2.4. Determination of Peroxide Value

The determination of peroxide value was carried out following the Commission Implementing Regulation (EU) No. 1348/2013 of December 2013 [23]. Briefly, an exactly weighted amount of about 1.5 g of olive oil was solubilized in 10 mL of chloroform into a ground glass-stoppered flask; then, 15 mL of acetic acid was added, followed by 1 mL of freshly prepared saturated aqueous solution of KI. The stopper was quickly inserted, then the mixture was shaken for 1 min and left for exactly 5 min in the dark at 25 °C. Then, 75 mL of distilled water was added and the liberated iodine was titrated with a sodium thiosulphate solution (0.01 M) using a starch solution (10 g/L aqueous dispersion) as an indicator. The peroxide value, expressed in milliequivalents of active oxygen per kilogram, was the result of an arithmetic mean between two determinations from the same sample of olive oil.

### 2.5. Determination of Biophenols by HPLC

The extraction and quantitative determination of the biophenolic minor polar compounds in olive oil samples was carried out according to the International Olive Council protocol with little modification [24]. Briefly, to an exactly weighted amount of about 2.0 g of olive oil in a screw-cap test tube, 1 mL of a 0.015 mg/mL solution of syringic acid in methanol/water (80:20 *v*/*v*) (internal standard) was added. The tube was sealed and shaken for 30 s, then 5 mL of a methanol/water solution (80:20 *v*/*v*) was added and the tube was sealed again, shaken for 1 min, sonicated in an ultrasonic extraction bath for 15 min, and finally centrifuged at 5000 rpm for 25 min. An aliquot of the supernatant was filtered through a 0.45 µm PVDF filter and injected (injection volume 10 µL) into a ZORBAX^®^ RRHT Eclipse Plus C18 (Santa Clara, CA, USA), 4.6 × 100 mm UHPLC column for the determination, eluted with H_2_O (0.2% H_3_PO_4_ *v*/*v*)/MeOH/ACN (96:2:2 → 50:25:25 over 40 min, 50:25:25 → 40:30:30 over 5 min, 40:30:30 → 0:50:50 over 15 min, and 0:50:50 for 10 min). The biophenol content, expressed in mg/kg, was calculated by measuring the sum of the areas of the related chromatographic peaks.

### 2.6. Determination of Tocopherols by HPLC

The determination of α-, β-, γ-, and δ-tocopherol was accomplished by following the UNI EN 12822:2014 p.to 5.4 protocol [25]. Briefly, an exactly weighted amount of about 2.0 g of olive oil was solubilized in 8 mL of acetone and injected (injection volume 5 µL) into a ZORBAX^®^ Eclipse XBD-C18, 4.6 × 150 mm, 5 µm analytical column, eluted with MeOH (0.1% acetic acid *v*/*v*)/AcOEt (100:0 for 3.5 min, 100:0 → 10:90 over 0.7 min, 10:90 for 2 min). The tocopherol content, expressed in mg/kg, was calculated by measuring the areas of the related chromatographic peaks.

### 2.7. Determination of Methyl Esters of Fatty Acids by GC

The determination of the methyl esters of fatty acids was carried out following the Commission Implementing Regulation (EU) 2015/1833 of 12 October 2015 Annex IV, with little modification [26]. Briefly, an exactly weighted amount of about 0.1 g of olive oil was solubilized in 2 mL of isooctane and then 100 µL of KOH solution in methanol (2 N) was added. The mixture was shaken for 1 min, left to stand for 2 min, and then 2 mL of NaCl (s.s.) was added. Finally, the mixture was centrifuged at 3500 rpm for 5 min and an aliquot of the supernatant was injected into an Agilent 8890 gas chromatograph (GC) system. Methyl esters of the fatty acid content, reported as the percentage area, was calculated by measuring the areas of the related chromatographic peaks.

### 2.8. Determination of Total Polyphenol Content by Folin–Ciocalteu Method

The determination of the total polyphenolic content was based on the work of Gutfinger [27] with little modification. Briefly, to a weighted amount of 1 g of olive oil, 1 mL of MeOH/H_2_O (80:20 *v*/*v*) solution was added. The mixture was shaken for 3 min and then centrifuged at 3500 rpm for 5 min. Then, 0.2 mL of supernatant was transferred to a flask containing 7.3 mL of H_2_O and 0.5 mL of Folin–Ciocalteau Reagent (FCR). Next, 2 mL of Na_2_CO_3_ aqueous solution (20% *w*/*v*) was added and the flask was sealed, shaken, and left to stand in the dark for at least 30 min; after this time, the sample was subjected to spectrophotometric analysis by measuring the extinction at 750 nm with a Cary 50 Scan^®^ UV–Vis spectrophotometer (Santa Clara, CA, USA) using caffeic acid as the standard for the preparation of the calibration curves. The total phenolic content, expressed as mg/kg of caffeic acid, was calculated as the arithmetic mean of three different determinations on the same olive oil sample.

### 2.9. Preparation of Polar Fraction Extracts

An exactly weighted amount of about 50 g of olive oil was dissolved in 50 mL of *n*-hexane and extracted three times with 30 mL of a MeOH/H_2_O solution (60:40, *v*/*v*). Each extract was washed once with 50 mL of *n*-hexane. The combined polar fractions were combined and the solvent was evaporated to dryness under vacuum at 40 °C [28].

### 2.10. ^1^H-NMR Analysis

NMR analysis was conducted on a Varian 300 MHz spectrometer (Milan, Italy) by diluting 5 mg of each sample in 600 µL of DMSO-*d*_6_. The assignment of the resonances was performed by analyzing ^1^H-NMR characteristics and by comparison with the literature [29,30,31].

### 2.11. UHPLC-HRMS 

HPLC analyses were performed on a Shimadzu UHPLC system, consisting of an LC-40B X3 solvent delivery module, an SPD-M40 photo diode array detector, a CTO-30A column oven, and an SIL-40C X3 autosampler. The instruments were coupled online with an LCMS-IT-TOF system (Shimadzu, Kyoto, Japan) equipped with an electrospray source (ESI) operated in negative mode. LC-MS data elaboration was performed using the LCMS solution^®^ software (Version 3.50.346, Shimadzu). For chromatographic analysis, a Luna^®^ Omega Polar C18 (L × I.D.: 100 × 2.1 mm × 1.6 µm, 100 Å) was employed (Phenomenex^®^, Bologna, Italy). The separation was carried out by employing H_2_O and ACN plus 0.1% CH_3_COOH as the mobile phases with the following gradient: 0–20 min, 10–90% B; 20–22 min, isocratic to 90% B; 22–24 min, 90–10%; and finally 4 min for column re-equilibration. The flow rate and column oven were set to 0.2 mL min^−1^ and 40 °C, respectively. Data acquisition was set in the range of 200–600 nm and chromatograms were monitored at 280 and 330 nm. Full-scan MS data were set to 100–1500 m/z and MS/MS experiments were conducted in the data-dependent acquisition process. Interface and curved desolvation line temperatures were set to 250 °C while nebulizing and drying gases (N_2_) were fixed at 1.5 and 10 L/min, respectively. For the prediction of the molecular formula, Formula Predictor software (Shimadzu) was used with the following settings: maximum deviation from mass accuracy of 10 ppm, fragment ion information, and nitrogen rule. The identification of compounds was based on accurate MS and MS/MS spectra, the retention times of available standards, and comparisons with the literature. Moreover, the following free online databases were consulted: ChemSpider (http://www.chemspider.com, accessed on 23 Januray 2022), SciFinder Scholar (https://scifinder.cas.org, accessed on 23 Januray 2022), and Phenol-Explorer (www.phenol-explorer.eu, accessed on 23 January 2022). For the quantitative analysis of flavones, luteolin and apigenin were selected as external standards and their amounts were expressed as milligrams per gram of extract.

### 2.12. Stable Isotope Ratio Analysis

The ^13^C/^12^C ratio in bulk Italian olive oils was measured in one run (and weighted around 0.5 mg) using an isotope ratio mass spectrometer (IRMS) (Isoprime Ltd., Stockport, UK) following total combustion in an elemental analyzer (VARIO CUBE, Elementar Analysensysteme GmbH, Dresden, Germany). The ^2^H/^1^H and ^18^O/^16^O ratios were measured in one go (around 0.5 mg) using an IRMS (Finnigan DELTA XP, Thermo Scientific, Milan, Italy) coupled with a pyrolyzer (Finnigan TC/EA high-temperature conversion elemental analyzer, Thermo Scientific). According to the IUPAC protocol, the values were denoted in delta in relation to the international V-PDB (Vienna-Pee Dee Belemnite) for δ^13^C and V-SMOW (Vienna-Standard Mean Ocean Water) and for δ^18^O and δ^2^H, according to the following general equation:
  δi E = (i RSA − i RREF)  i RREF
where i is the mass number of the heavier isotope of element E (for example, ^13^C); RSA is the respective isotope ratio of a sample (such as for C: number of ^13^C atoms/number of ^12^C atoms or as approximation ^13^C/^12^C); RREF is the respective isotope ratio of the internationally recognized reference material. For δ^13^C, the samples were analyzed using a single working standard for normalization and calibrated against NBS-22 fuel oil (IAEA-International Atomic Energy Agency, Vienna, Austria), IAEA-CH-6 sucrose, and USGS 40 (U.S. Geological Survey, Reston, VA, USA). We did not use a calibration curve for δ^13^C as suggested by IUPAC [32] because as we used a single standard with a value similar to that of the samples. The data determined using a single anchoring point or two or three anchoring points were not significantly different [33]. The δ^2^H and δ^18^O values of defatted protein were calculated against USGS 84 (Sicilian olive oil standard δ^2^H = −140.4 ± 3.1 ‰ and δ^18^O = +26.38 ± 0.5‰) and USGS 86 (tropical Vietnamese peanut oil standard, δ^2^H = −207.4 ± 4.5‰ and δ^18^O = +18.76 ± 1.03‰) through the creation of a linear equation and adoption of the “comparative equilibration procedure” [34]. One control sample was routinely included in each analytical run to check the system performance and we obtained very repeatable results over the 2 month running period. Measurement uncertainty rates, expressed as one standard deviation when measuring a sample 10 times, were ≤2‰ for δ^2^H, 0.3‰ for δ^18^O, and 0.2‰ for δ^13^C. 

### 2.13. Antioxidant–Anti-Inflammatory Activity of EVOO Extracts

#### 2.13.1. Cell Culture and Treatments

Human colorectal adenocarcinoma CaCo-2 cells were cultured in high-glucose Dulbecco’s modified Eagle’s medium (Corning, NY, USA) supplemented with 10% FBS (Merck, Darmstadt, DE, Germany), 100 U/L penicillin, 100 μg/mL streptomycin (Gibco, ThermoFisher Scientific, Waltham, MA, USA), and 1% of non-essential amino acids (ACL006, Microtech, Pozzuoli, NA, Italy). All cell cultures were performed at 37 °C in 5% CO_2_ and 95% air. The experiment cells were treated for 24 h with EVOO extracts **OE_05**, **OE_10**, and **OE_15** suspended in DMSO (vehicle) at a concentration of 20 μg/mL and then exposed to glucose oxidase (GO) (cat 195196, MP Biomedicals™) at a concentration of 0.5 U/mL for 1 h. After the different treatments and exposure, samples were collected at the indicated timepoints for subsequent immunofluorescence staining, rt-PCR (Table S1), and AmplexRed assay analysis. 

#### 2.13.2. Cytotoxicity Study (MTT Assay)

To evaluate the cytotoxicity of olive oil extracts and to choose the treatment dose, the cell viability test with MTT 3-(4,5-dimethylthiazol-2-yl)-2,5-diphenyltetrazolium bromide assay was performed on CaCo-2 cells and carried out as previously described [35]. Cells were grown in 96-well plates at a density of 2 × 10^4^ cells/well in 200 μL of media and then pre-treated for 24 h with different concentrations of the olive oil extracts **OE_05**, **OE_10**, and **OE_15**, ranging from 2 to 50 μg/mL. After complete removal of the treatment to avoid any color interference, 50 μL of serum-free media and 50 μL of MTT solution (0.5 mg/mL) were added and incubated for 3 h. The insoluble purple formazan crystals were then dissolved in 100 μL of DMSO at 37 ◦C for 15 min. After shaking, the solution absorbance was measured using the Multiskan GO microplate spectrophotometer (Thermofisher Scientific, Milan, Italy) at 570 nm, using 630 nm as a reference wavelength. The results are expressed as percentages of cell viability.

#### 2.13.3. AmplexRed Assay

H_2_O_2_ production rate was evaluated using the Amplex Red–horseradish peroxidase (HRP) (P8375 (Merck, Darmstadt, DE, Germany) method in the media of CaCo-2 cells. As previously reported [36], cells were pre-treated for 24 h with olive oil extracts **OE_05**, **OE_10**, and **OE_15** 20 μg/mL and then exposed for 1 h to GO 0.5 U/mL. After GO exposure, media were replaced with fresh DMEM HG 10% FBS and then collected directly after exposure (T0) and 30 min (T30′) and 1 h (T1) post-exposure. Briefly, 10 μL of media for each sample was added to the reaction mixture, where in the presence of horseradish peroxidase (HRP), H_2_O_2_ reacted with the Amplex^®^ Red reagent (A12222, ThermoFischer Scientific, Waltham, MA, USA), resulting in the formation of the red fluorescent resorufin product. The content of H_2_O_2_ was determined through a 4P logistic regression curve by comparing the fluorescence at Ex/Em 531/595 nm with that of the H_2_O_2_ standard curve. Readings were performed using a Victor3 microplate reader (PerkinElmer, Inc., Waltham, MA, USA). The calibration curve was assessed using H_2_O_2_ solutions as the standard, and the H_2_O_2_ production is expressed in μM. 

#### 2.13.4. Immunofluorescence Staining

CaCo-2 cells were grown on coverslips at a density of 0.8 × 10^5^ cells/ mL in 24-well plates. After 24 h of pre-treatment with 20 μg/mL of **OE_05**, **OE_10**, and **OE_15** olive oil extracts, cells were exposed to 1 h of GO 0.5 U/mL and then collected right after the end of GO exposure (T0) and 1 h post-exposure (T1). The immunofluorescence staining was assessed as previously described [37]. Briefly, cells were washed twice in PBS, fixed in 4% paraformaldehyde (PFA) in PBS for 10 min at room temperature, and then permeabilized with 0.25% of Triton X-100 in PBS for 10 min RT. After the blocking step in 2% BSA in PBS for 45 min at RT, coverslips were then incubated with primary antibody Nrf2 (Santacruz, sc-365949) 1:50 in 0.25% BSA/PBS and NF-κB (8242, Cell Signaling, Danvers, MA, USA) 1:300 in 0.25% BSA/PBS overnight at 4 °C. The day after, samples were incubated for 1 h with fluorochrome-conjugated secondary antibodies (A11003 Alexa Fluor 546, A11008 Alexa Fluor 488) in PBS at BSA 0.25%. Nuclei were stained with 1 μg/mL DAPI for 5 min after the removal of the secondary antibody. Coverslips were mounted onto glass slides using PermaFluor Aqueous Mounting Medium (TA-06-FM ThermoFisher Scientific, Waltham, MA, USA) and examined using the Axio Imager A2 microscope equipped with a Leica DFC350 FX camera (Carl Zeiss s.p.a, Milan, Italy) at 40× magnification. Images were quantified using ImageJ software.

#### 2.13.5. RNA Extraction and Quantitative Real Time PCR (qRT-PCR)

CaCo-2 cells were seeded at a density of 4 × 10^5^ cells in 6 wells plate, pre-treated for 24 h with the extracts **OE_05**, **OE_10** and **OE_15**, and then exposed to GO 0.5 U/mL for 1 h. Samples were collected 6 h (T6) post GO treatment. Total RNA was extracted from CaCo-2 cells using the Ribospin (304–150, GeneAll) and Riboclear plus (313–150, GeneAll) kits, according to the manufacture’s protocol. RNA concentration was measured using the Multiskan GO microplate spectrophotometer (Thermofisher Scientific, Milan, Italy) with MicroDROP plate (ThermoFisher Scientific, Waltham, MA, USA). cDNA was generated from 1 μg of total RNA, using the IScript reverse Transcription Supermix for RT-qPCR kit (1708841, BioRad, Milan, Italy). To evaluate the mRNA levels of NQO1, GPx1, COX-2 and IL-6 genes (Table S1) quantitative real-time PCR was performed using the KAPA SYBR FAST qPCR Master Mix (2×) kit (KR0389-v10.16, KAPA BIOSYSTEMS) on a ViiA^TM^ 7 Real-Time PCR System (Thermofisher Scientific, Waltham, MA, USA) following the manufacturer’s protocol. Gene expression was quantified by obtaining the number of cycles to reach a predetermined threshold value in the intensity of the PCR signal (CT value). Beta actin was used as reference gene, while samples were compared using the relative cycle threshold (CT). After normalization, quantitative relative gene expression was calculated by the 2^−∆∆Ct^ method [38].

### 2.14. Vasoactivity Assessments of EVOO Extracts

#### 2.14.1. Animals

All the study procedures were in strict accordance with the European Union Guidelines for the Care and the Use of Laboratory Animals (European Union Directive 2010/63/EU) and approved by the Animal Care and Ethics Committee of the University of Siena and Italian Department of Health (7DF19.N.TBT). Male Wistar rats (331 ± 11 g) were purchased from Charles River Italia (Calco, Milan, Italy) and maintained in an animal house facility at 25 ± 1 °C with a 12:12 h dark/light cycle with access to standard chow diet and water ad libitum. Animals were anaesthetized with an isoflurane (4%) and O_2_ gas mixture using Fluovac (Harvard Apparatus, Holliston, MA, USA), decapitated, and exsanguinated. The thoracic aorta was immediately isolated and placed in physiological solution (namely modified Krebs–Henseleit solution (KHS)) and prepared as detailed below.

#### 2.14.2. Preparation of Rat Aortic Rings

The thoracic aorta was gently cleaned of adipose and connective tissues and cut into 3-mm-wide rings. These were mounted in organ baths between two parallel L-shaped stainless steel hooks, one fixed in place and the other connected to an isometric transducer [39]. Rings were allowed to equilibrate for 60 min in KHS (composition in mM: 118 NaCl, 4.75 KCl, 1.19 KH_2_PO_4_, 1.19 MgSO_4_, 25 NaHCO_3_, 11.5 glucose, 2.5 CaCl_2_, gassed with a 95% O_2_–5% CO_2_ gas mixture to create a pH of 7.4) under a passive tension of 1 g. During this equilibration period, the solution was changed every 15 min. The isometric tension was recorded using a digital PowerLab data acquisition system (PowerLab 8/30; ADInstruments). Ring viability was assessed by recording the response to 0.3 µM phenylephrine (Sigma Chimica, Milan, Italy) and 60 mM KCl. Where needed, the endothelium was removed by gently rubbing the lumen of the ring with forcep tips. This procedure was validated by adding 10 µM acetylcholine (Sigma Chimica, Milan, Italy) at the plateau of phenylephrine-induced contraction: a relaxation greater than 70% or less than 10% denoted the presence or absence of functional endothelium, respectively [40]. 

#### 2.14.3. Effect of EVOO Extracts on Phenylephrine-Induced Contraction

The effects of EVOO extracts, added cumulatively, were assessed on 0.3 μM phenylephrine-induced contraction in either endothelium-intact or -denuded rings. Sodium nitroprusside (100 µM; Riedel-De Haën AG, Seelze-Hannover, Germany) was used to prove smooth muscle functional integrity at the end of each concentration–response curve. The response to phenylephrine was taken as 100%.

### 2.15. Statistical Analysis

For each of the variables tested, analysis of variance (one-way ANOVA) was used followed by Tukey’s post-hoc test. Statistical significance was considered at *p* < 0.05. Data are expressed as means ± SD of duplicate determinations obtained in at least 3 independent experiments. Analyses of data, statistical analyses, and significance analyses, as measured by Student’s t test for unpaired samples (two tailed), were accomplished using GraphPad Prism version 5.04 (GraphPad Software Inc., San Diego, CA, USA). Data are reported as means ± SEM; *n* is the number of rings analyzed (indicated in parentheses), isolated from at least three animals. The pharmacological response to each EVOO extract is described in terms of the IC_50_.

## 3. Results and Discussion

We decided to analyze the qualitative profiles of EVOO samples in order to select the most interesting sample in terms of polyphenol content for further biological evaluation.

### 3.1. Acidity and Peroxides Analysis

Acidity and peroxide analyses were carried using standard protocols, as described in the Materials and Methods section. As reported in Table 1, all the samples showed very low acidity values, expressed as percentages of oleic acid, ranging from 0.08 to 0.15%. Similarly, peroxide values, expressed as meq O_2_/kg, appeared to be <10, far below the limit that the Italian legislation set as the maximum amount allowed to be labeled as EVOO (20 meq O_2_/kg). Taken together, these values demonstrated the high quality of the fruits and the observation of good practices during the harvest and the milling processes. 

### 3.2. Determination of Methyl Esters of Fatty Acids by GC

This analysis was performed as described above to characterize the percentages of oleic and linoleic acids present in the EVOO samples. Table 1 lists the values of oleic acid (a monounsaturated ω-9 fatty acid) and linoleic acid (a polyunsaturated ω-6 fatty acid), being the most represented components of olive oil triglycerides (up to 83% of the total fatty acid content). We decided to concentrate our attention on these fatty acids, as it is now widely accepted that they can exert interesting health-promoting effects, such as anti-inflammatory, antioxidant, and cardio-protective effects [41,42]. These fatty acids presented similar values among all samples.

### 3.3. Tocopherols

Tocopherols represent the vitamin E content of olive oil, and they are known to play a synergistic antioxidant effect together with biophenols. Moreover, a supranutritional intake of tocopherols has been reported to be beneficial in cardiovascular diseases, cancer, inflammation, and neurodegenerative diseases [43]. For these reasons, we decided to evaluate the tocopherols content of the 18 EVOO samples by following the above-mentioned protocol. As reported in Table 1, all samples display total contents of tocopherols ranging between 164 and 298 mg/kg, with the noteworthy exception of **O_10**, which possesses a remarkable value of 446 mg/kg.

### 3.4. Total Phenolic Content and Biophenols

In order to evaluate the biological properties of the EVOO extracts, we performed two different analyses to elucidate the phenolic content. In particular, we applied an HPLC protocol to quantify the content of biophenols, such as the natural and oxidized derivatives of oleuropein and ligstroside, lignans, flavonoids, and phenolic acids [24]. As reported in Table 1, biophenol contents ranged from 227 mg/kg to 664 mg/kg, with good values shown by **O_01** and **O_08**, constituted by three typical Tuscan *cv*., and by **O_15** and **O_10**, constituted only by the rare *Minuta di Chiusi* and the more widespread *Leccino cv.*, respectively. Furthermore, the total phenolic content (TPC) was measured using the Folin–Ciocalteu method, a recognized protocol for the quantitative determination of total phenolic compounds. Generally, all EVOO samples displayed good TPC values ranging from 406 to 713 mg/kg, with **O_01**, **O_03**, **O_08**, **O_10**, **O_14**, and **O_15** being the richest samples (600 to 700 mg/kg). Based on their TPC and biophenols values, we selected three samples, namely **O_05**, **O_10**, and **O_15**, to obtain their hydroalcoholic extracts, which were used to evaluate the biological properties of the phenolics present in representative EVOOs from Siena area. In particular, **O_15** was selected due to its high TPC value (682 mg/kg) and because it is entirely produced from *Minuta di Chiusi* olives. Although **O_10** was selected mainly for its high TPC value (713 mg/kg), it is noteworthy that it came from *Leccino monocultivar*, a *cv*. that is widespread in Italy. On the other hand, **O_05** was chosen as a representative sample of intermediate TPC values, but also for its miscellaneous composition in terms of *cv.*

### 3.5. Isotopic Ratio Mass Spectrometry

It is widely known that EVOO, and Italian EVOO above all, is one of the main food products subjected to fraud. For this reason, we decided to submit some of the olive oil samples to the stable isotope ratio analysis, which by means of isotope ratio mass spectrometry (IRMS) can assay the content of the stable isotopes of some elements (H, C, O in this case) that are related to agricultural practices and pedoclimatic features of the specific geographical area where the olive trees are grown. Specifically, the isotopic ^13^C/^12^C, ^18^O/^16^O, and ^2^H/^1^H ratios change according to different factors, such as latitude, altitude, climate conditions, and distance from the sea [44]. In Table 2, the above-mentioned isotopic ratios for 6 EVOO samples are reported, with the future aim of creating a database of the stable isotopic ratios of local olive oil samples from different *cv.*, which can be applied to certify the origin and quality of the products.

### 3.6. Extraction and Chemical Characterization via ^1^H NMR and HRMS

As described in the material and methods section, starting from about 50 g of EVOO, we obtained different amounts of polar extracts. From **O_05** we obtained 66 mg of extract **OE_05**, from **O_10** we obtained 71 mg of extract **OE_10**, and from **O_15** we obtained 63 mg of extract **OE_15**. To verify the outcome of the extraction and to appreciate the chemical composition of the extracts, we performed ^1^H NMR experiments to identify the peculiar signals given by the main different components. The results shown in Table 3 demonstrate the presence of the main distinguishable phenols, such as tyrosol, hydroxytyrosol, and *p*-coumaric acid; secoiridoids, such as oleuropein and oleochantal; and lignans, such as 1-acetoxypinoresinol, among others. In the aliphatic region of the spectra, these polar components signals are hidden underneath the fatty acids and mono- and diacylglycerols (chemical shifts of δ 2.80–2.50 and δ 3.65–3.20 ppm) (Figure S1), suggesting a certain degree of co-extraction of some apolar compounds [29].

**Figure 2:**
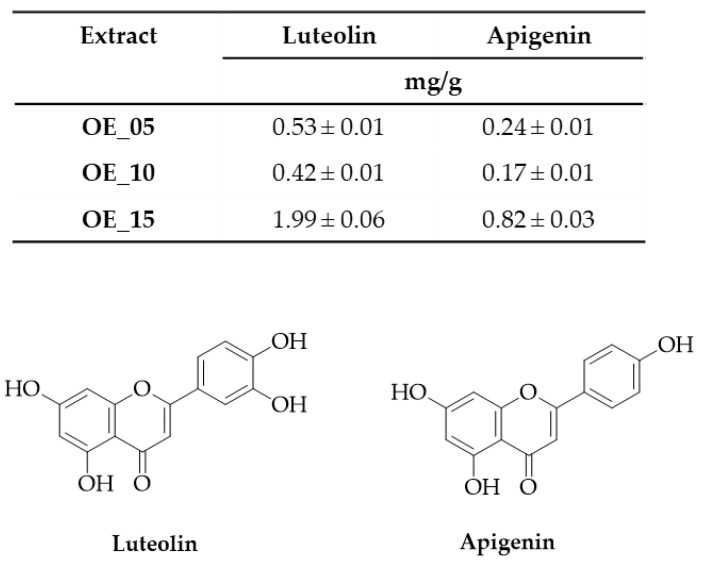
Quantification of luteolin and apigenin in EVOO extracts (OE).

The HRMS analysis showed the presence of those compounds identified by NMR experiments. Nevertheless, the UHPLC analysis (Table 4) revealed the presence of the same compounds in all the samples (Figures S2–S4 and Table S2). The principal compounds identified were oleacinic acid, oleocanthalic acid, various isomers of oleuropein, and ligstroside (aglycone and glycosides forms), although in particular two polyphenols, oleuropein and luteolin, were present in high amounts, which were experimentally calculated as reported in Figure 2.

### 3.7. Evaluation of Protective Effects of **OE_05**, **OE_10**, and **OE_15** on CaCo-2 Cells

#### 3.7.1. MTT Assay

Upon arrival, EVOO extracts were suspended in 100 μL of DMSO and stored at 4 °C, avoiding direct light. The MTT assays for **OE_05**, **OE_10**, and **OE_15** were performed on CaCo-2 cells to evaluate the non-toxic treatment dose. Cells were treated for 24 h with different concentration of EVOO extracts (2, 5, 10, 20, and 50 μg/mL) and DMSO at different doses (vehicle 1 and vehicle 2), suspended in completed DMEM high-glucose media.

**Figure 3:**
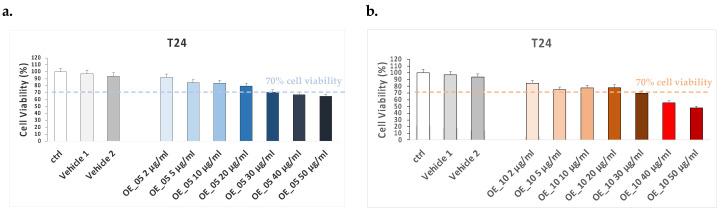
CaCo-2 cell viability evaluated by MTT assay after 24 h of pre-treatment with olive oil compound extracts **OE_05** and **OE_10** at different doses ranging from 2 to 50 μg/mL. (**a**) Vehicle 1 represents the DMSO concentration of **OE_05** or **OE_10** 2 μg/mL solution. (**b**) Vehicle 2 represents DMSO concentration of **OE_05** or **OE_10** 50 μg/mL solution. Ctrl represents untreated cells. Data are given as means ± SD, representative of three independent experiments with at least three technical replicates each time.

As shown in Figure 3, the doses that led to a slight cell viability decrease were in the range of 30 to 50 μg/mL (less than 70% of cell viability), whereas the other doses did not display any significant differences in cell viability compared to control cells (ctrl). Hence, since the 20 μg/mL concentration was the highest dose not displaying toxicity on CaCo-2 cells, we decided to use EVOO extracts at this concentration to perform the experiment and ensure a possible protective effect.

#### 3.7.2. Antioxidant Properties of EVOO Extracts

**Figure 4:**
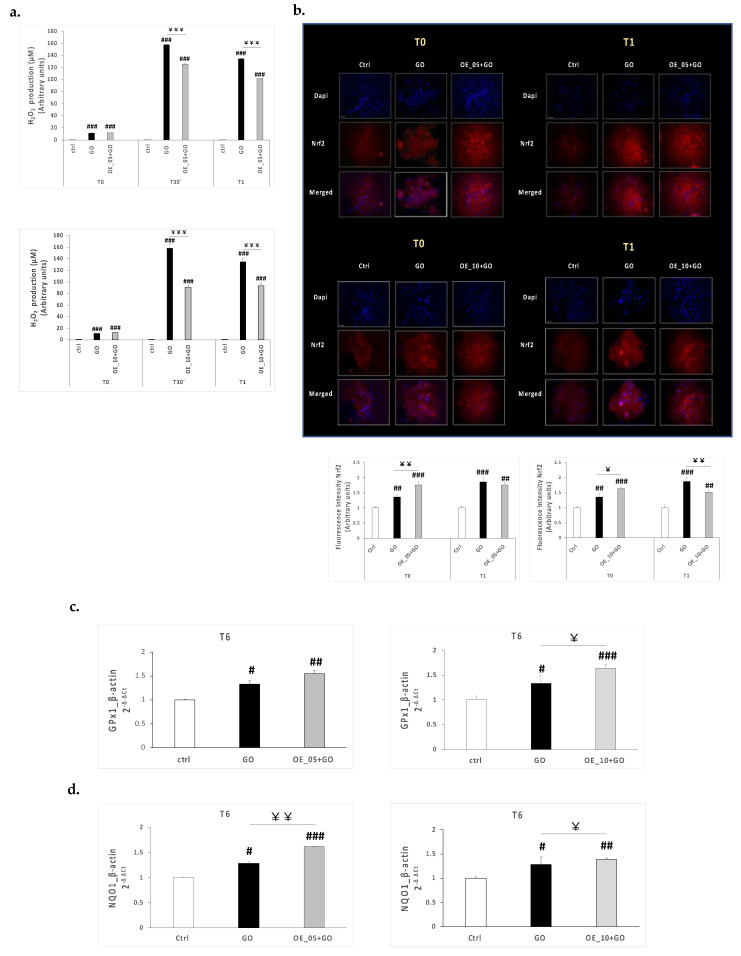
(**a**) H_2_O_2_ levels in media of CaCo-2 cells treated with 20 μg/mL **OE_05** or **OE_10** for 24 h and exposed to GO 0.5 U/mL for 1 h (T0, T30′, T1). (**b**) Immunofluorescence staining of DAPI (blue) and Nrf2 (red), 0 and 1 h post-GO exposure in CaCo-2 cells pre-treated with 20 μg/mL of **OE_05** or **OE_10** for 24 h. Original magnification at 40x; scale bar = 40μm. The immunofluorescent signal was semiquantified by using ImageJ software (National Institutes of Health, Bethesda, MD) and the quantification graphs are displayed in the bottom panel. Transcript levels of GPx1 (**c**) and NQO1 (**d**) measured using qRT-PCR 6 h post-GO exposure in CaCo-2 cells pre-treated with EVOO extracts for 24 h. Data are the results of the averages of at least three different experiments, # *p* < 0.05, ## *p* < 0.01, ### *p* < 0.001 GO ± **OE_05** or **OE_10** vs. ctrl and ￥ *p* < 0.05, ￥￥ *p* < 0.01, ￥￥￥ *p* < 0.001 GO + **OE_05** or **OE_10** vs. GO as assessed by one-way ANOVA.

To evaluate the antioxidant properties of EVOO extracts, we triggered CaCo-2 cells with the oxidoreductase enzyme glucose oxidase (GO), which catalyzes the oxidation of glucose in hydrogen peroxide (H_2_O_2_), a known oxidative mediator. After pre-treating CaCo-2 cells with 20 μg/mL **OE_05**, **OE_10**, or **OE_15**, we assessed an AmplexRed assay to measure the levels of H_2_O_2_ produced over 1 h of GO 0.5 U/mL treatment. As depicted in Figure 4a, GO exposure induced higher levels of H_2_O_2_ production at all selected timepoints. Even though **OE_05** and **OE_10** did not completely abrogate the H_2_O_2_ production compared to control cells, they could reduce the levels of H_2_O_2_ induced by GO. To test whether the reduction of the H_2_O_2_ production found in CaCo-2 cells was due to the ability of EVOO extracts to induce an antioxidant response responsible for counteracting the GO-induced oxidative impairment, we assessed by immunofluorescence staining the possible activation of the nuclear factor erythroid 2-related factor 2 (Nrf2). Indeed, Nrf2 activation can control the transcription of several genes involved in the cellular antioxidant response. As depicted in Figure 4b, the GO insult induced a slight increase in Nrf2 expression levels right after GO exposure (T0) and even more evidently after 1 h (T1). Interestingly, both EVOO extracts led to significant increases in Nrf2 expression levels upon GO insult, especially at T0, and these increases were still evident 1 h after GO exposure, even if they were lower compared to GO exposure alone. Since the activation of Nrf2 can trigger the transcription of several genes involved in the antioxidant response of the cell to restore cell homeoastasis [45], we decided to measure the transcriptional expression levels of two of the main genes under the control of Nrf2, glutathione peroxidase 1 (GPx1) and NAD(P)H dehydrogenase (quinone) 1 (NQO1). We found that GO upregulated the mRNA expression levels of both GPx1 (Figure 4c) and NQO1 (Figure 4d) 6 h post exposure and that the cells pre-treated with either **OE_05** or **OE_10** and exposed to GO displayed much higher mRNA expression levels of these two genes compared to GO-exposed cells. Since both GPx1 and NQO1 are required in the detoxification process of the cells, helping in reducing the production of radical species, their increased mRNA expression could suggest the ability of EVOO extracts to stimulate the antioxidant response of the cells and make the cells more promptly respond to the eventual oxidative insult. Indeed, although the olive oil compounds did not completely prevent the oxidative impairment induced by the GO insult as suggested by the presence of high H_2_O_2_ levels even after the pre-treatment, the compounds showed an ability to modulate the antioxidant response of the cells by increasing the Nrf2 expression and the transcriptional levels of Nrf2-related genes GPx1 and NQO1 upon GO insult.

#### 3.7.3. EVOO Extracts Showed Anti-Inflammatory Properties against Glucose Oxidase Insult

Nowadays, oxinflammation is a well-known phenomenon characterized by the cross-talk of oxidative and inflammatory features that can influence each other to promote a more severe cellular homeostasis impairment in a variety of conditions [46,47,48,49].

**Figure 5:**
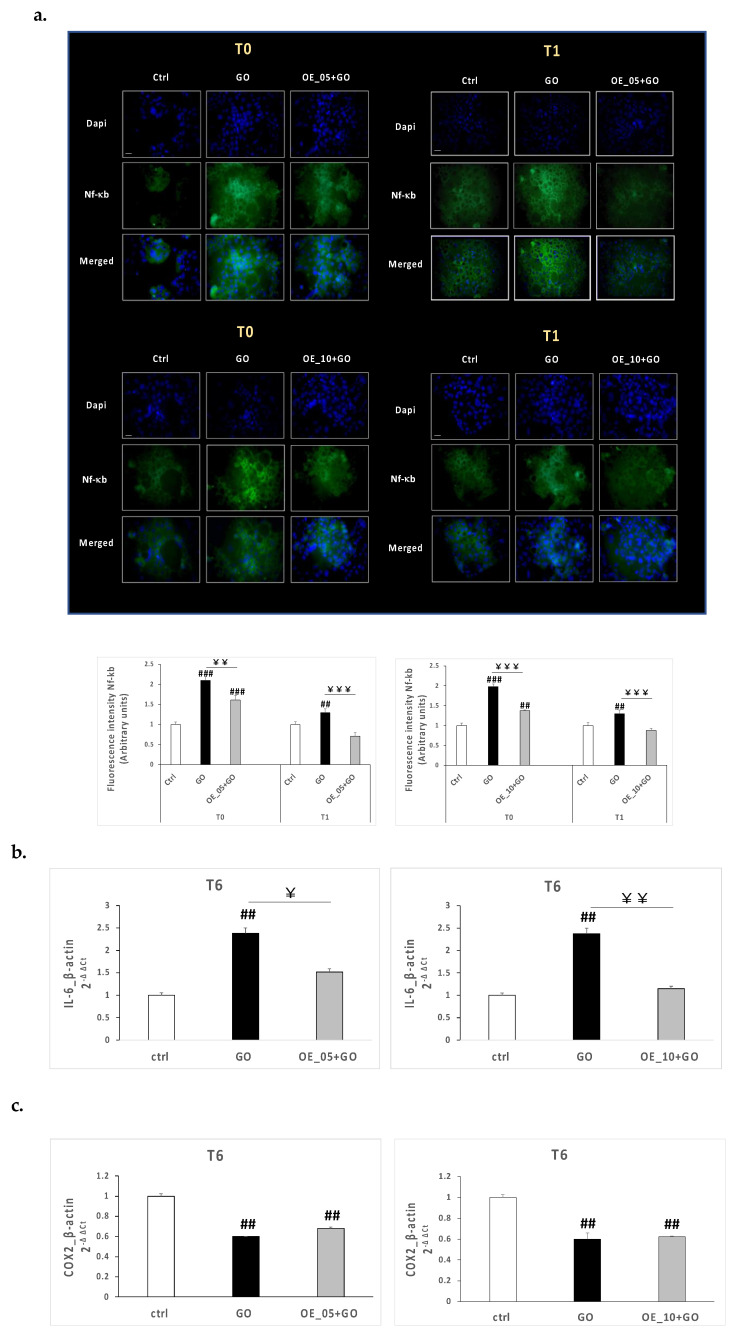
(**a**) Immunofluorescence staining of DAPI (blue) and NF-κB (green) in CaCo-2 cells treated with 20 μg/mL of **OE_05** or **OE_10** for 24 h and exposed to GO 0.5 U/mL for 1 h. Timepoints at 0 and 1 h post-exposure (T0, T1). Original magnification at 40x; scale bar = 40μm. The immunofluorescent signal was semiquantified using ImageJ software (National Institutes of Health, Bethesda, MD) and the quantification graphs are displayed in the bottom panel. Transcript levels of IL-6 (**b**) and COX2 (**c**) measured using qRT-PCR 6 h post-GO-exposure in CaCo-2 cells pre-treated with EVOO extracts for 24 h. Data are the results of the averages of at least three different experiments, ## *p* < 0.01, ### *p* < 0.001 GO ± **OE_05** or **OE_10** vs. ctrl and ￥ *p* < 0.05, ￥￥ *p* < 0.01, ￥￥￥ *p* < 0.001 GO + **OE_05** or **OE_10** vs. GO as assessed by one-way ANOVA.

To evaluate whether the altered oxidative status induced by the GO insult in CaCo-2 cells could affect and activate also an inflammatory response and whether the EVOO extracts **OE_05**, **OE_10**, and **OE_15** could display anti-inflammatory properties, we measured several markers involved in the inflammatory response. Hence, immunofluorescence staining and rt-PCR analysis were carried out to investigate the expression levels of one of the most activated transcription factors, nuclear factor kappa-light-chain-enhancer of activated B cells (NF-κB), as well as the mRNA expression levels of some NF-κB related genes such as Interleukin-6 (IL-6) and cyclooxygenase 2 (COX2), which are usually upregulated during inflammatory status. As depicted in Figure 5a, GO exposure significantly increased NF-κB expression levels either at 0 or 1 h post-exposure, suggesting the activation of an inflammatory response upon the oxidative insult due to a cross-talk between oxidative and inflammatory pathways. Of note, **OE_05** and **OE_10** reduced the expression of NF-κB at both timepoints, completely restoring the NF-κB basal-level expression 1 h after the end of GO exposure, as in control cells. In addition, **OE_05** and **OE_10** pre-treatment completely abrogated the increased IL-6 mRNA expression levels induced 6 h after GO exposure, confirming how the presence of specific substances, including flavones apigenin and luteolin, contributed to the anti-inflammatory properties [50] of the two EVOO extracts (Figure 5b). Regarding COX2, the gene did not turn out to be modulated by GO insult, as shown in Figure 5c.

These data are in line with **OE** polyphenol content data. In fact, other studies demonstrated how EVOO phenolics were able to inhibit TLR4 (Toll-like receptor 4) activity, which in turn inhibited NF-κB signaling, reducing the secretion of pro-inflammatory cytokines and chemokines by PBMCs (peripheral blood mononuclear cells) such as IL-1β and CXCL1 (chemokine C-X-C motif Ligand 1) at the molecular level, as well as IL-6 [51].

On the other hand, EVOO extracts derived from different cultivars showed potent anti-inflammatory properties thanks to a marked upregulation of the antioxidant enzymes heme oxygenase 1, NADPH quinone oxidoreductase 1, thioredoxin reductase 1, and glutathione reductase [9].

Furthermore, the phenolic compounds present in EVOO extracts showed promising anti-inflammatory properties; in particular, luteolin is able to inhibit H_2_O_2_-induced oxidative stress, activating the AMPK and Nrf2 pathways [52], while apigenin is able to reduce the production of inflammatory markers such as TNF-α, TGF-β, IL-1β, and IL-6, with significant inhibition of the active caspase-3 pathway and the pro-apoptotic Bax protein [53].

### 3.8. Effects of EVOO Extracts on Phenylephrine-Induced Contraction in Rat Thoracic Aorta Rings

**Figure 6:**
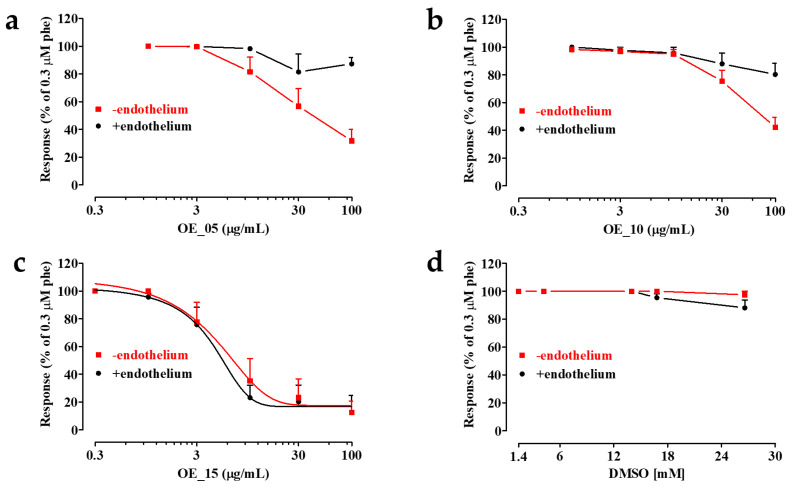
Effect of the EVOO extracts on phenylephrine-induced contraction of rat aorta rings. Concentration-response curves of (**a**) **OE_05**, (**b**) **OE_10**, and (**c**) **OE_15** on endothelium-denuded (-endothelium) or endothelium-intact (+endothelium) preparations pre-contracted by 0.3 µM phenylephrine (phe). (**d**) The effects of the vehicle DMSO are shown. In the ordinate scale, the response is reported as a percentage of the initial tension induced by phenylephrine, taken as 100%. Data are means ± SEM (*n* = 5–9).

The vasorelaxant activity of EVOO extracts was assessed on α_1_ adrenergic-receptor-mediated vascular smooth muscle contraction. As shown in Figure 6, **OE_05**, **OE_10**, and **OE_15** caused a concentration-dependent relaxation of endothelium-denuded rings contracted by 0.3 μM phenylephrine, with IC_50_ values of 41.8 µg/mL (estimated), 75.7 µg/mL (estimated), and 6.4 ± 1.6 µg/mL (*n* = 5). In a second series of experiments, the three extracts were assessed on rings with an intact endothelium. **OE_15** reverted phenylephrine-induced contraction, with an IC_50_ value that was not significantly different from that recorded in preparations devoid of endothelium (4.1 ± 1.9 µg/mL, *n* = 6; *p* = 0.3866). However, both **OE_05** and **OE_10** showed markedly lower vasorelaxant activity levels, accounting for a mere 20% relaxation of the active muscle tone (Figure 6a,b).

These data demonstrate that **OE_05** and **OE_10** stimulate the release of endothelium-derived contracting factors, which are yet to be characterized. In endothelium-denuded rings, in fact, myorelaxant activity occurred at lower concentrations as compared to endothelium-intact specimens. Contrary to **OE_05** and **OE_10**, **OE_15**, besides being the most effective extract, also showed a vasorelaxant activity that was not influenced by the presence of an intact endothelium. This supports the hypothesis that the three extracts are capable of directly targeting the smooth muscle cells and activating vasorelaxant mechanisms, thereby ensuring a persistent decrease in active muscle tone, a phenomenon worthy of note. In fact, it is reasonable to think that this endothelium-independent vasorelaxant activity can counteract the reduced NO synthesis that characterizes a dysfunctional endothelium occurring in cardiovascular diseases, such as hypertension [54,55,56].

Taking into account the chemical composition of the extracts, their vasoactivity is likely due to the presence of apigenin and luteolin which act as potent K_Ca_1.1 channel stimulators [57,58,59], as well as oleuropein and tocopherols, although the latter are known to act mainly in an endothelium-dependent manner [60,61].

However, apigenin and luteolin are also effective Ca_V_1.2 channel current stimulators [50], thereby leading to vessel contraction. Therefore, it is conceivable that the combined actions of all the components present in the extracts may be the main driver of vasorelaxation, as recently demonstrated in our laboratory [59].

Another important issue that should be taken into account is the extensive metabolism affecting polyphenol bioavailability and causing a partial loss of or even masking their bioefficacy [62,63]. These metabolites, however, can be locally converted back to the parent compound, for example through the activity of glucuronidases, thereby preserving the same activity observed in vitro [64].

## 4. Conclusions

In this work, we investigated autochthonous Tuscany EVOO *cv.* by evaluating their qualitative properties as source of fats. Then, selected EVOOs were subjected to extraction in order to obtain polar extracts, which were further analyzed. In particular, they were chemically characterized via ^1^H-NMR and UHPLC-HRMS analyses, showing the presence of luteolin and apigenin as representative flavones, as well as oleuropein, ligstroside, and pinoresinol, which are known to be present in EVOO polar fractions. These three extracts were then analyzed as anti-inflammatory phytocomplexes in CaCo-2 cells exposed to glucose oxidase. They reduced the inflammatory stimuli by modulating Nrf2 and NF-κB pathways, probably due to the presence of flavone compounds. Furthermore, they promoted vasorelaxation in rat aorta rings contracted by phenylephrine. In conclusion, we showed new potential nutraceutical applications of EVOO polar extracts obtained from autochthonous Tuscany *cv.* grown in the Siena area.

## Figures and Tables

**Table 1:** Quantification of the main components in EVOO samples.

EVOO Samples	Cultivars	Acidity (% Oleic Acid)	Oleic Acid (%)	Linoleic Acid (%)	TPC (mg/kg)	Biophenols (mg/kg)	Tocopherols (mg/kg)	Peroxides (meq O_2_/kg)
**O_01**	*Leccino* (80%) *Frantoio* (10%) *Moraiolo* (10%)	0.15	75.48	6.28	703	664	298	7.70
**O_02**	*Leccino* (10%) *Frantoio* (80%) *Moraiolo* (10%)	0.11	76.68	5.86	517	405	164	8.00
**O_03**	*Minuta* (70%) *Greggiolo* (30%)	0.12	75.72	6.01	632	451	260	7.20
**O_04**	*Leccino* (100%)	0.11	76.92	5.75	467	321	228	6.80
**O_05**	*Greggiolo* (50%) *Leccino* (10%) *Moraiolo* (10%) *Frantoio* (10%) *Morello* (20%)	0.11	77.04	5.95	406	314	221	6.50
**O_06**	*Leccino* (50%) *Frantoio* (50%)	0.14	77.54	5.63	474	293	228	8.40
**O_07**	*Leccino* (60%) *Frantoio* (30%) *Moraiolo* (10%)	0.10	76.18	5.99	547	345	223	9.30
**O_08**	*Leccino* (50%) *Frantoio* (50%)	0.11	75.96	6.07	627	560	270	7.30
**O_09**	*Frantoio* (40%) *Moraiolo* (30%) *Leccino* (30%)	0.13	77.34	5.78	465	372	167	8.50
**O_10**	*Leccino* (100%)	0.11	76.45	5.73	713	477	446	5.30
**O_11**	*Leccino* (100 %)	0.08	76.47	5.68	444	290	291	6.20
**O_12**	*Leccino* (60%) *Frantoio* (20%) *Moraiolo* (20%)	0.09	77.20	5.75	586	405	207	5.70
**O_13**	*Leccino* (80%) *Frantoio* (10%) *Moraiolo* (10%)	0.09	77.14	5.40	410	227	238	7.10
**O_14**	*Leccino* (10%) *Frantoio* (70%) *Moraiolo* (20%)	0.09	75.75	5.98	602	434	254	6.30
**O_15**	*Minuta* (100%)	0.13	75.53	6.34	681	553	239	4.90
**O_16**	*Leccino* (80%) *Frantoio* (10%) *Moraiolo* (10%)	0.12	75.83	6.16	540	380	216	6.20
**O_17**	*Leccino* (30%) *Frantoio* (30%) *Moraiolo* (30%) Others (10%)	0.10	77.09	5.66	440	243	222	7.90
**O_18**	*Frantoio* (80%) *Leccino* (10%) *Moraiolo* (10%)	0.10	76.44	5.98	529	385	261	7.00

**Table 2:** Stable isotopic ratios of six EVOO samples.

EVOO Samples	Cultivars	Altitude (m asl)	^13^C/^12^C (δ^13^C) (‰ vs. V-PDB)	^18^O/^16^O (δ^18^O) (‰ vs. V-SMOW)	^2^H/^1^H (δ^2^H) (‰ vs. V-SMOW)
**O_05**	*Greggiolo* (50%) *Leccino* (10%) *Moraiolo* (10%) *Frantoio* (10%) *Morello* (20%)	450	−30.2‰	23.2‰	−146‰
**O_08**	*Leccino* (50%) *Frantoio* (50%)	350	−30.9‰	22.8‰	−149‰
**O_09**	*Frantoio* (40%) *Moraiolo* (30%) *Leccino* (30%)	300	−30.1‰	23.2‰	−150‰
**O_10**	*Leccino* (100%)	300	−30.5‰	22.1‰	−148‰
**O_15**	*Minuta* (100%)	330	−29.6‰	23.5‰	−149‰
**O_16**	*Leccino* (80%) *Frantoio* (10%) *Moraiolo* (10%)	600	−30.5‰	23.2‰	−150‰

**Table 3:** ^1^H NMR qualitative analysis of the major components in EVOO extracts **OE_05**, **OE_10**, and **OE_15**.

Compound	Assignment	^1^H (ppm)	Multiplicity	Samples		
				OE_05	OE_10	OE_15
Oleuropein	CHOH	9.49	m	+	+	+
Oleocanthal	CHO	9.23	s	+	+	+
*p*-Coumaric acid	CH=CH	7.54	d	+	+	+
Tyrosol (total)	-	7.08–6.96	not assigned	+	+	+
Hydrotyrosol	-	6.54–6.41	not assigned	+	+	+
Carotenoids (total)	CH	6.68	m	+	+	+
Luteolin	C6-H	6.18	m	−	+	−
Ligstroside	-	4.20–4.00	not assigned	+	+	+
Pinoresinol	OCH_3_	3.75	s	+	−	+
1-Acetoxypinoresinol	OCH_3_	3.76	s	−	+	+
Campesterol	CH_3–_18	0.70	s	+	+	+
β-Sitosterol	CH_3–_18	0.68	s	+	+	+

Note: (+) presence, (−) absence.

**Table 4:** UHPLC-HRMS qualitative analysis of the major components in EVOO extracts **OE_05**, **OE_10**, and **OE_15**.

Peak	Retention Time (min)	[M-H]^-^ (*m/z*)	MS/MS	Error (ppm)	Proposed Compound	Molecular Formula
00	3.07 ± 0.01	153.0556	123.4023; 95.4011	−0.65	3-Hydroxytyrosol	C_8_H_10_O_3_
0	4.46 ± 0.03	137.0602	112.0045	−4.37	Tyrosol	C_8_H_10_O_2_
1	6.47 ± 0.03	335.1093	199.0564; 155.0665	−8.95	Oleacinic acid	C_17_H_20_O_7_
2	8.77 ± 0.02	319.1168	181.0452; 199.0551	−5.95	Oleocanthalic acid	C_17_H_20_O_6_
3	9.41 ± 0.02	285.0410	175.0382; 199.0373	2.10	Luteolin	C_15_H_10_O_6_
4	9.57 ± 0.11	357.1368	136.0393; 342.0891	7.00	Pinoresinol	C_20_H_21_O_6_
5	10.14 ± 0.19	377.1205	275.0829; 149.0197; 139.0074	0.7	Oleuropein aglycone	C_19_H_21_O_8_
6	10.32 ± 0.04	377.1205	275.0831; 149.0199; 139.0076	0.9	Oleuropein aglycone Isomer II	C_19_H_21_O_8_
7	10.51 ± 0.02	269.0414	225.0481; 150.0228; 117.0289	5.7	Apigenin	C_15_H_10_O_5_
8	10.67 ± 0.16	377.1205	275.0829; 149.0197; 139.0074	−4.4	Oleuropein aglycone Isomer III	C_19_H_21_O_8_
9	10.90 ± 0.10	377.1201	275.0842; 149.0174; 139.0010	−5.4	Oleuropein aglycone Isomer IV	C_19_H_21_O_8_
10	11.42 ± 0.19	361.1250	291.0780; 259.0926	−4.1	Ligstroside-aglycone	C_19_H_22_O_7_
11	12.21 ± 0.02	361.1242	291.0801; 259.0865	−9.0	Ligstroside-aglycone Isomer II	C_19_H_22_O_7_
12	12.63 ± 0.02	361.1271	291.0808; 259.0909	−4.2	Ligstroside-aglycone Isomer III	C_19_H_22_O_7_

## Data Availability

The data presented in this study are available in the article and supplementary materials.

## Supplementary Materials

The following are available online at https://www.mdpi.com/article/10.3390/antiox11030437/s1, Table S1. Primer Sequences. Figure S1. Representative ^1^H-NMR spectrum of **OE_15**. Figure S2. **OE_05** ʎ:280 nm. Figure S3. **OE_10** ʎ:280 nm. Figure S4. **OE_15** ʎ:280 nm. Table S2. Method validation parameters for quantitation by RP-UHPLC-PDA of flavones in olive oil extracts
