# Supplementary material for: Extra Virgin Olive Oil Extracts of Indigenous Southern Tuscany Cultivar Act as Anti-Inflammatory and Vasorelaxant Nutraceuticals
Source: Antioxidants (Basel). 2022 Feb 22;11(3):437. doi: 10.3390/antiox11030437 (PMC8944769; doi:10.3390/antiox11030437)

antioxidants

Supplementary Material for:

Extra Virgin Olive Oil Extracts of Indigenous Southern Tuscany Cultivar Act as Anti-inflammatory and Vasorelaxant Nutraceuticals

Luca Pozzetti [1], Francesca Ferrara [2], Ludovica Marotta [1], Sandra Gemma [1], Stefania Butini [1], Mascia Benedusi [2], Fabio Fusi [1], Amer Ahmed [3], Serena Pomponi [4], Stefano Ferrari [5], Matteo Perini [6], Anna Ramunno [7], Giacomo Pepe [7], Pietro Campiglia [7], Giuseppe Valacchi [8,9,10], Gabriele Carullo [1,*] and Giuseppe Campiani [1,*]

Contents

Table S1. Primer Sequences
Figure S1. Representative ^{1}H-NMR spectrum of **OE_15**
Figure S2. OE_05 ʎ:280 nm
Figure S3. OE_10 ʎ:280 nm
Figure S4. OE_15 ʎ:280 nm
Table S2. Method validation parameters for quantitation by RP-UHPLC-PDA of flavones in olive oil extracts

Table S1. Primer sequences

Gene	Forward sequence	Reverse sequence
GPx1	CAACCAGTTTGGGCATCAG	TCTCGAAGAGCATGAAGTTGG
NQO1	CTGATCGTACTGGCTCACTC	AACAGACTCGGCAGGATAC
IL-6	TAGGACTGGAGATGTCTGAGGCT	GACCGAAGGCGCTTGTGGA
COX-2	TTCAAATGAGATTGTGGGAAAATT	ATATCATCTCTGCCTGAGTATCTT
β-actin	ATTGCCGACAGGATGCAGA	AGTACTTGCGCTCAGGAGGA

Figure S1. Representative ^{1}H-NMR spectrum of OE_15

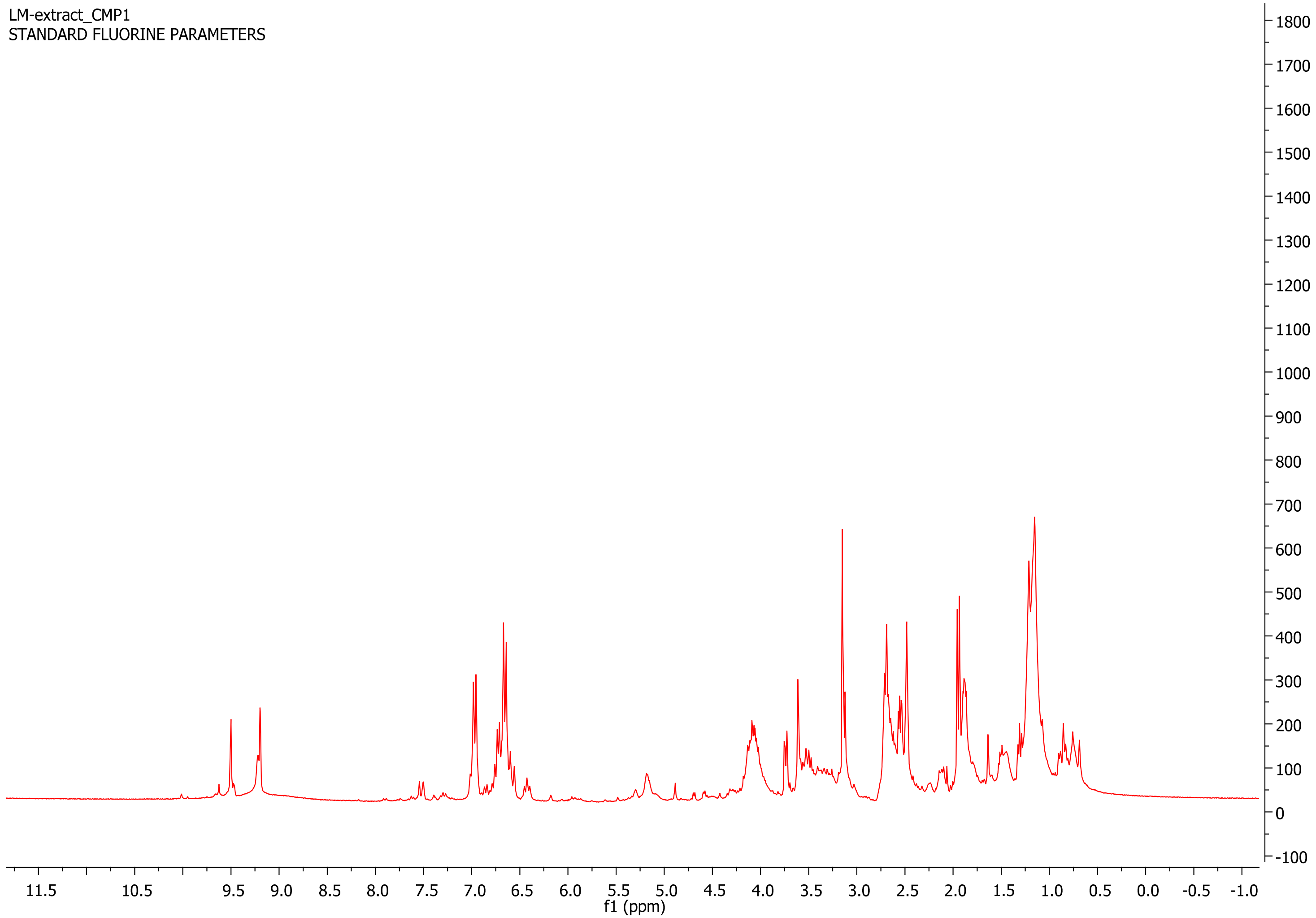

Figure S2. OE_05 λ:280 nm

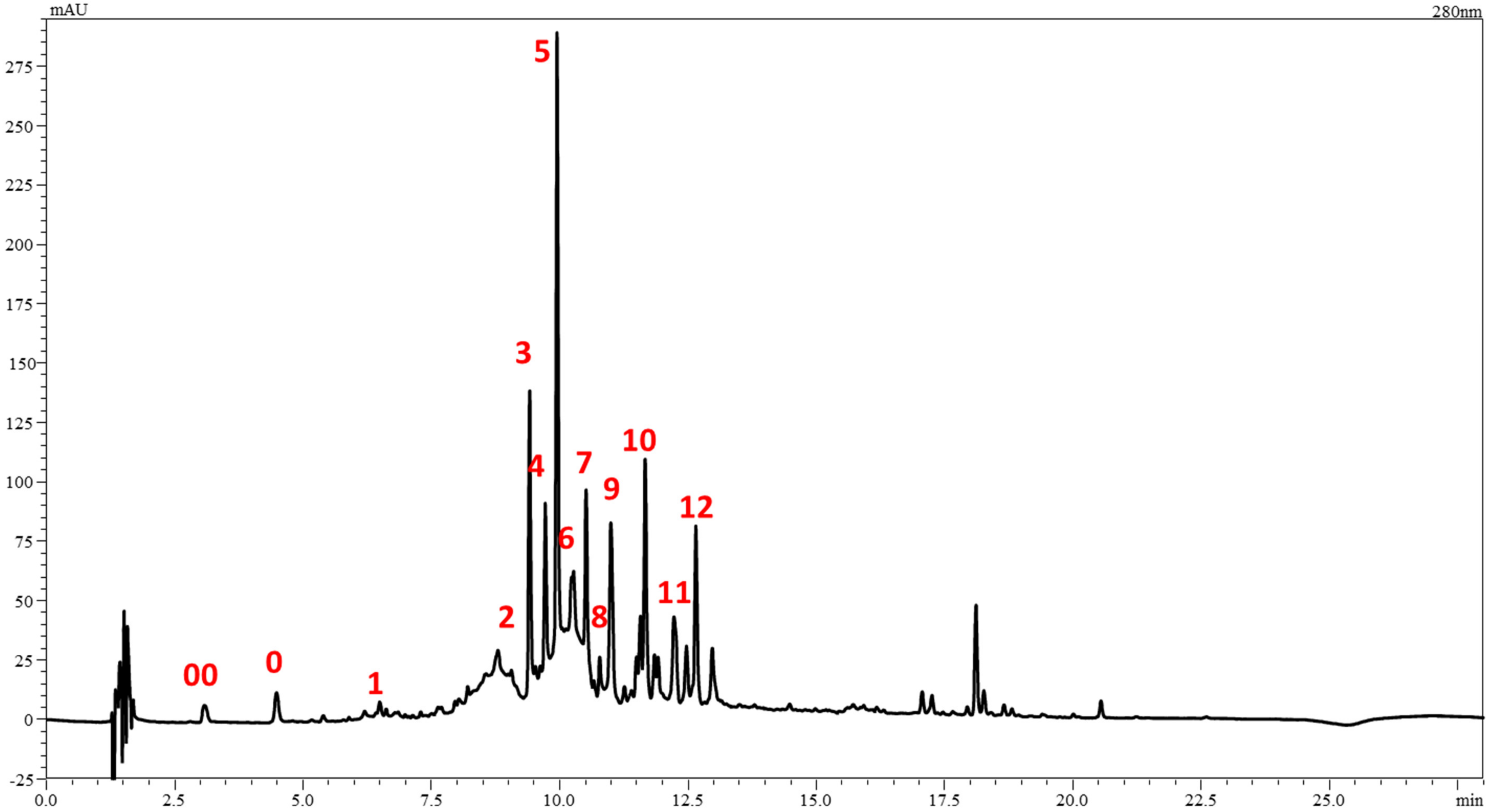

Figure S3. OE_10 λ:280 nm

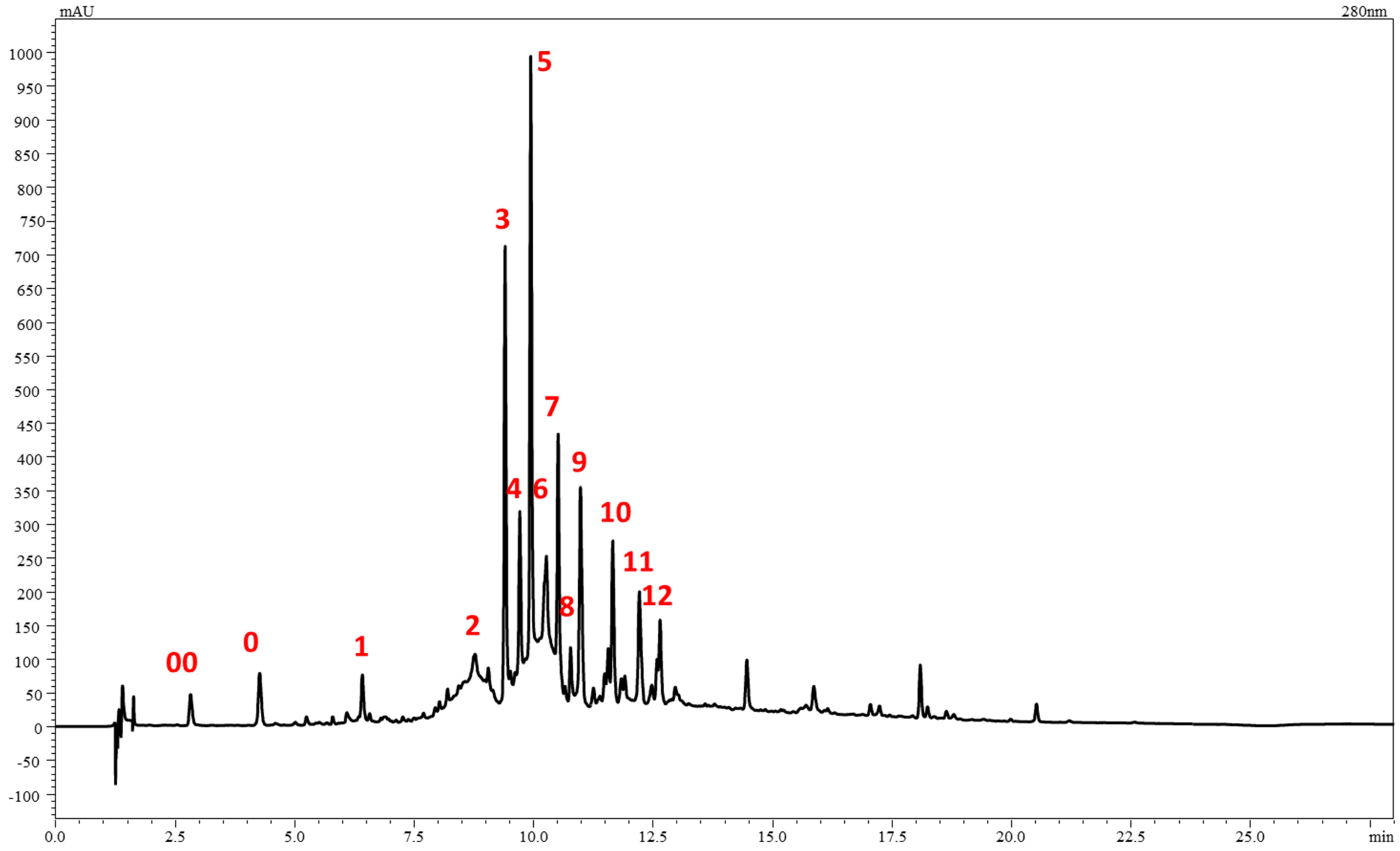

Figure S4. OE_15 *λ*:280 nm

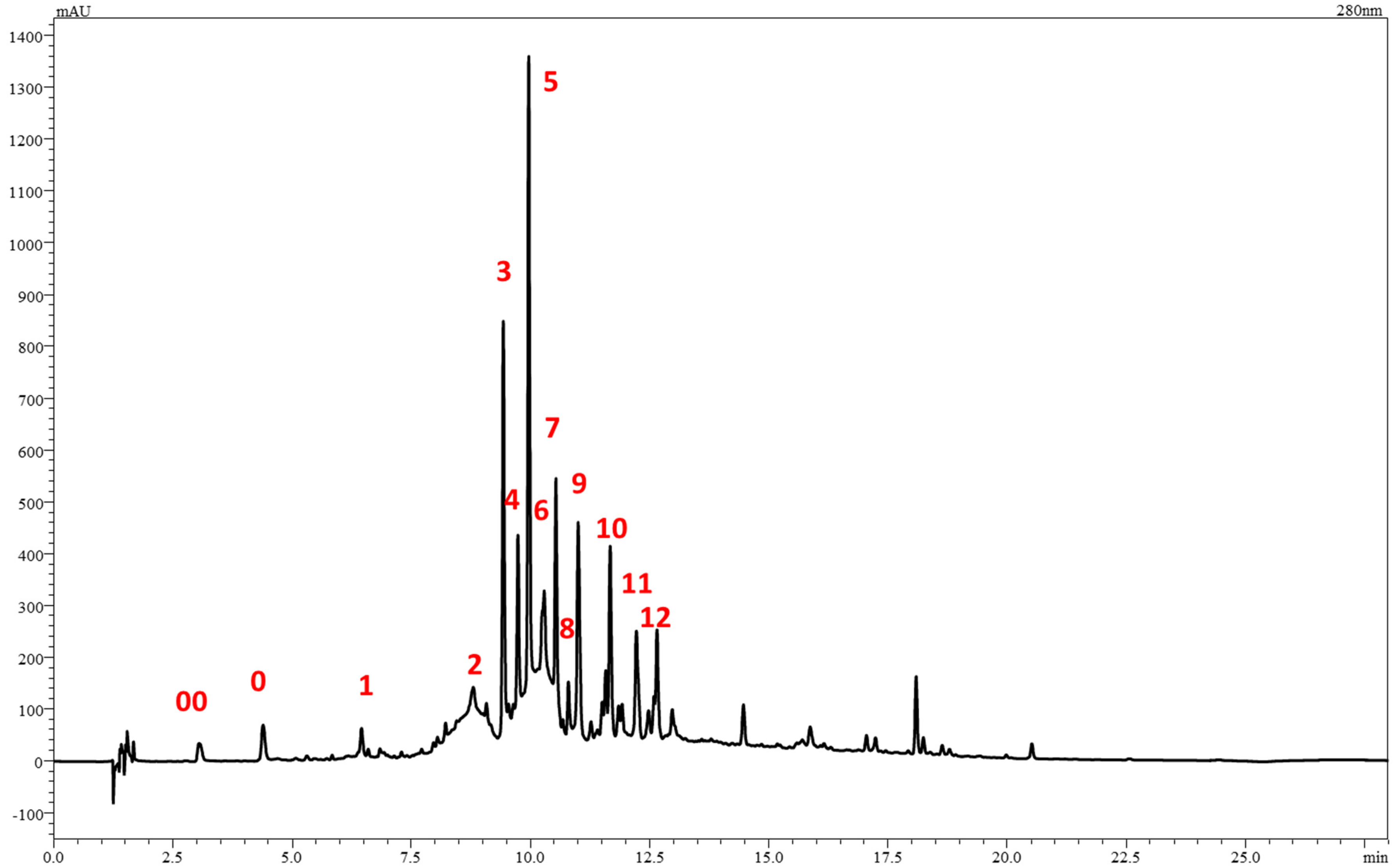

Table S2. Method validation parameters for quantitation by RP-UHPLC-PDA of flavones in olive oil extracts

Parameters		Luteolin		Apigenin	
Retention time (min) (n = 4)		9.41 ± 0.01		10.51 ± 0.01	
Regression equation		y = 0.000036x + 0.059537		y = 0.000029x – 0.159557	
Correlation coefficient (R^2)		≥ 0.9999		≥ 0.9999	
LOD (µM)		0.06		0.01	
LOQ (µM)		0.17		0.04	
	Analyte concentration (µM)	RSD (%)	Accuracy (%)	RSD (%)	Accuracy (%)
Intraday (n = 3)	10	0.2%	99.7%	0.1%	102.1%
	20	0.4%	98.7%	0.1%	100.9%
	50	0.2%	98.7%	0.1%	101.7%
Interday (n = 3)	10	0.4%	98.8%	0.2%	100.9%
	20	0.1%	98.9%	0.1%	101.4%
	50	0.2%	98.8%	0.1%	102.5%

Y: concentration of tested compounds; X: peak area at 330 nm

LOQ: 10 × standard deviation of the response/slope of calibration curve

LOD: 3.3 × standard deviation of the response/slope of calibration curve

Extract	Luteolin (mg/g)	Apigenin (mg/g)
OE_05	0.53 ± 0.01	0.24 ± 0.01
OE_10	0.42 ± 0.01	0.17 ± 0.01
OE_15	1.99 ± 0.06	0.82 ± 0.03

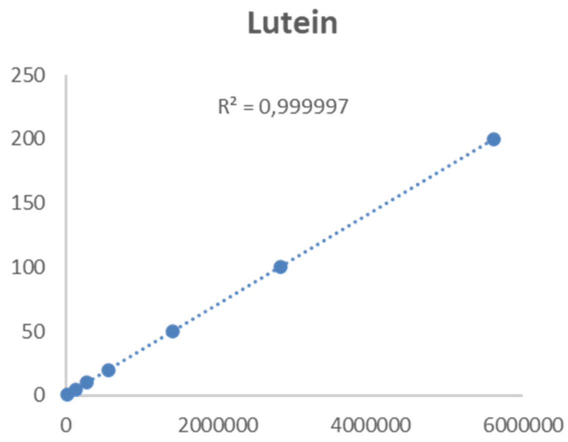

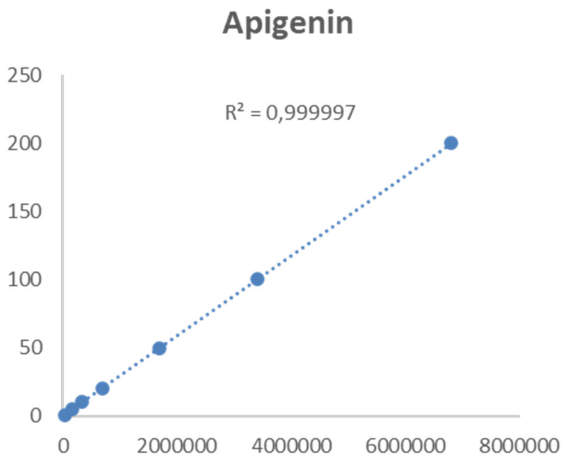

Supplement: Supplementary file 1 [file antioxidants-11-00437-s001.zip › antioxidants-1589823-supplementary.pdf]
